# Visual recognition of honeybee behavior patterns at the hive entrance

**DOI:** 10.1371/journal.pone.0318401

**Published:** 2025-02-25

**Authors:** Tomyslav Sledevič, Artūras Serackis, Dalius Matuzevičius, Darius Plonis, Gabriela Vdoviak

**Affiliations:** 1 Department of Electronic Systems, Faculty of Electronics, Vilnius Gediminas Technical University – VILNIUS TECH, Vilnius, Lithuania; PMAS Arid Agriculture University: PMAS-Arid Agriculture University Rawalpindi, PAKISTAN

## Abstract

This study presents a novel method for automatically recognizing honeybee behavior patterns at the hive entrance, significantly contributing to beekeeping and hive management. Utilizing advanced YOLOv8 models for detection and segmentation, our approach analyzes various aspects of bee behavior, including location, direction, path trajectory, and movement speed within a designated area on the hive’s landing board. The system effectively detects multiple bee activities such as foraging, fanning, washboarding, and defense, achieving a mean detection accuracy of 98% and operating at speeds of up to 36 fps, surpassing state-of-the-art methods in both speed and accuracy. Key contributions include the development of a comprehensive dataset with 7200 frames from eight beehives, the introduction of the first known research focused on recognizing bee behavior patterns through visual analysis at the hive entrance, and a comparative evaluation of various object detection and tracking algorithms tailored for bee detection and behavior recognition. Our findings indicate that this method enhances monitoring capabilities for beekeepers while reducing the need for manual inspections, thereby minimizing disturbances to the bees. By analyzing spatial trajectories and occurrence density maps, the proposed framework provides robust identification of overlapping behaviors, facilitating timely interventions when necessary. This work lays the groundwork for future automated monitoring systems aimed at improving hive health and productivity.

## Introduction

Tracking the condition of beehives is essential for beekeepers to maintain the health and productivity of their bee colonies. By monitoring hive conditions, beekeepers can detect potential problems early and take preventive or corrective actions, thereby reducing the risk of colony collapse and ensuring the continued pollination, production of honey, beeswax, and other bee products [1]. Several methods can be employed to monitor hive conditions, including non-intrusive visual surveillance [2,3], acoustic and vibration monitoring [4,5], as well as tracking temperature, humidity, and weight [6]. Utilizing these and other monitoring techniques enables beekeepers to promptly address any issues and ensure the ongoing success of their colonies [7].

The entrance of the hive is a vital component of the bee colony’s life, as it allows the colony to breathe and take away unwanted debris. It also provides beekeepers with insights into the colony’s health and potential problems. One of the simplest ways to monitor the condition of the beehive is through visual observation of the entrance. By watching the activity at the hive entrance, beekeepers can track the number of bees coming and going, assess their activity levels, and look for signs of pests or disease. Observing bee behavior both inside and outside the hive can help determine whether the bees are active and healthy. For example, frequent flights in and out of the hive, the collection of pollen and nectar, and wing fanning to regulate temperature and humidity are all indicators of a healthy hive. Overall, monitoring bees at the entrance provides valuable information about the hive’s condition and aids beekeepers in making informed decisions about hive management [7].

Understanding the internal condition of the hive is crucial for beekeepers to prevent financial losses. By observing the entrance of the hive, beekeepers can assess the colony’s condition without opening the hive and disturbing the bees [8]. If an experienced beekeeper can recognize bee behavior patterns simply by observing the entrance, then a machine should be capable of doing the same. This would eliminate the need for beekeepers to periodically approach each hive in the apiary for visual inspections; instead, a machine equipped with an image processing module for bee behavior pattern recognition could perform this task. Such a system would assist beekeepers in monitoring hive conditions, enabling them to quickly detect and respond to any changes in hive activity or behavior. Additionally, it could provide real-time alerts for unusual bee behavior, signaling when beekeeper intervention is necessary.

Several types of honeybee behavior can be visually identified at the entrance of the beehive, including foraging, robbing, guarding, defending, swarming, temperature regulation (fanning), washboarding, and orientation flights of young bees. It is important to note that while some of these behaviors may be observable at the hive entrance, determining the exact behavior or role of a particular bee often requires closer observation. Additionally, the prevalence of certain behaviors may vary depending on factors such as the time of day, weather conditions, and other circumstances [9]. In general, bees will spend varying amounts of time at the entrance ramp based on their specific roles and the needs of the colony. Therefore, to accurately identify bee behavior at the hive entrance, it is essential to have a comprehensive dataset that encompasses all potential behavior patterns.

In this work, we developed a reliable and efficient method for automatically recognizing bee behavior patterns through visual analysis, providing valuable insights into hive conditions. The method is based on the latest YOLOv8 models trained for bee detection and segmentation. It considers various aspects of bee behavior, including their locations, directions, path trajectories, and movement speeds within a restricted zone on the hive’s landing board. The method detects different bee activities, such as foraging, fanning, washboarding, and defense. The achieved detection speed outperforms that of state-of-the-art approaches, and the accuracy exceeds the average reported in related literature. Our contributions can be summarized as follows:

- Collected, annotated, and publicly provided dataset [10] for:
  - Bee detection: 7200 frames from 8 beehives.
  - Direction estimation based on segmentation: 2300 cropped bees.
  - Tracking and behavior classification: 4 classes with 17162 tracks across 21946 frames.
- Presented the first known research on recognizing bee behavior patterns based on the analysis of bee movements at the entrance of the beehive.
- Conducted a comparative evaluation of object detection and tracking algorithms for bee detection, direction estimation, tracking, and behavior pattern recognition.

The proposed work represents the first step toward developing a long-term automated monitoring system that processes visual data to extract behavioral statistics and identify significant events at the beehive entrance. The optional visualization module allows users to view predicted behavior classes in real-time or periodically capture frames for reporting purposes.

The method utilizes bee location, orientation, path trajectory, and movement speed within a restricted zone on the hive’s landing board, ensuring that both spatial and temporal dynamics are captured for behavior recognition. By analyzing spatial data, the system can track the location and movement patterns of individual bees within the hive environment. This enables the identification of specific behaviors based on the trajectory and positioning of bees in relation to the hive entrance. Temporal information complements this by providing insights into the timing and duration of these activities, allowing for a more comprehensive understanding of bee behavior over time. The integration of spatial and temporal data enhances the accuracy of activity recognition by enabling the system to differentiate between overlapping behaviors that may occur simultaneously. For example, bees may exhibit both fanning and foraging behaviors in close proximity; thus, understanding their movement speed and direction is crucial for accurate classification. Furthermore, this approach allows for the detection of subtle changes in behavior patterns that may indicate shifts in hive health or environmental conditions, facilitating timely interventions by beekeepers. Utilizing spatial and temporal information not only improves the robustness of behavior identification but also provides a richer dataset for analysis. This leads to better monitoring capabilities, enabling beekeepers to maintain optimal hive conditions and enhance overall colony health and productivity.

## Related works

Visual monitoring of honeybee colonies can be implemented in several ways: by observing bee activity at the hive entrance, tracking the trajectories of bees in unconstrained flight conditions, or sensing bee activity within the beehive.

### Monitoring bees at the entrance

Majewski et al. [2] developed a method based on machine learning models to predict the remaining time of foraging activity and to prevent bee poisoning from crop spraying before the end of foraging. This method takes into account bee activity on the hive’s landing board, weather conditions, and the time until sunset, utilizing data collected from IoT systems during the beekeeping season. Bee detection was performed with an average precision of 95% using Mask R-CNN with ResNet50 as the backbone network. A total of 180 images were used for training and validation, and density maps of bee occurrence were employed to estimate the total number of bees. The authors achieved an RMSE of 23 to 26 minutes in predicting the remaining time of bee foraging activity.

Mukherjee and Kulyukin [3] implemented an algorithm to measure incoming, outgoing, and lateral honeybee traffic at the hive entrance. They employed digital particle image velocimetry to quantify directional honeybee traffic levels and applied the dynamic time warping algorithm to assess the similarity scores of bee traffic curves. Bees were detected and counted based on motion vectors computed between two consecutive video frames.

Ngo et al. [11] developed an automated system for monitoring honeybee activity. This system utilized an observation box attached to the hive, equipped with an integrated webcam to capture frames. To track multiple bees, the researchers applied the Kalman filter along with the Hungarian algorithm. Recently, they expanded their study by incorporating a CNN-based bee detection approach [12]. Specifically, they trained a tiny YOLOv3 model to detect multiple bees, both with and without pollen grains. The model achieved a real-time image processing speed of 25 fps on an embedded Jetson TX2 GPU system, with a classification accuracy of 94%. The datasets used for training and testing consisted of 3000 and 500 images, respectively.

Two studies by Babić et al. [13] and Stojnić et al. [14] developed algorithms to detect pollen-bearing honeybees and classify them upon entering the hive. The proposed methods involved image segmentation followed by feature extraction using SIFT and VLAD descriptors on the segmented images. Classification was performed using an SVM classifier, which achieved an accuracy of 89% when trained with 100 images and operated at approximately 1 fps on a Raspberry Pi [13]. By increasing the training dataset to 800 images, the classification accuracy improved to 92%, and the segmentation process achieved a nearly 6 fps rate [14]. Nguyen et al. [15] introduced a dataset for detecting pollen-bearing honeybees at the hive entrance, comprising 60826 annotated instances across 2051 images. Their proposed method integrates YOLOv5 and Faster R-CNN models, achieving a precision of 99% and demonstrating its potential for enhancing automated beehive monitoring.

Kulyukin and Mukherjee [16] developed an algorithm to estimate bee traffic levels at the hive entrance based on motion detection and image classification. Each detected motion region is classified by a custom CNN into two classes: "bee" or "not bee", with accuracy ranging from 89% to 94%.

Yang and Collins [17] utilized Faster R-CNN with the VGG16 network to detect pollen grains in individual bee images. The bee detection model was then combined with a Kalman filter-based tracking model, allowing each flying bee tracked in successive video frames to be identified as carrying pollen or not. The authors trained the network on 1000 images and achieved a classification accuracy of 94% on 400 test images.

Tu et al. [18] developed a system based on Raspberry Pi to analyze the behavior of bees at the entrance of the hive. This system effectively counts honeybees, identifies their positions, and measures their in-and-out activity. Bee detection relies on background subtraction and statistical analysis. The system achieves an accuracy of 98.7% for bee counting, 95.3% for measuring in-activity, and 88.8% for measuring out-activity. It saves 30 seconds of video every 10 minutes at a frame rate of 5 fps and processes this footage offline over the next 567 seconds, resulting in a relatively low effective frame rate.

The orientation of the bee can be estimated by tracking its posture [19]. Rodriguez et al. [20] presented an approach that detects five body parts: the tip of the abdomen, thorax, head, left antenna, and right antenna, with defined connections between them. The pose detector architecture employs a CNN with a feature extraction backbone and two branches for pose detection. During the inference stage, a greedy algorithm is used to detect keypoints of body parts and group them into skeletons, providing a trajectory of the bee’s pose over time. The average precision for body part detection reaches 99% on a custom dataset collected at the hive entrance, which utilized an artificial ramp colored blue to improve detection accuracy.

### Monitoring colony inside the hive

Bozek et al. [21] implemented a method to track the entire honeybee colony using high-resolution video of the natural honeycomb inside the hive. The authors adapted a CNN-based segmentation architecture to automatically identify bee and brood cell positions, body orientations, and within-cell states. They achieved a 10% error in body width positioning, a $10^{\circ }$ error in orientation, and a true positive rate exceeding 90%.

Gernat et al. [22] presented a method that combines CNNs with barcode-based tracking to accurately identify specific honeybee behaviors within the hive. They used barcode information to pinpoint image regions relevant to the behavior of interest, which were then analyzed with a CNN to confirm the occurrence of the behavior. The authors designed detectors for liquid transfer between bees and for egg-laying. Their approach achieved 67% higher sensitivity and an 11% lower error rate compared to the best previously used detector.

Bilik et al. [23] investigated object detection techniques for the visual diagnosis of Varroa destructor parasitic mites in honeybees using images of natural honeycomb. They compared YOLO and SSD object detectors alongside the Deep SVDD anomaly detector. Utilizing a custom dataset of 600 ground-truth images of healthy and infected bees in various scenes, the detectors achieved the highest F1 score of up to 87.4% for detecting infected bees and up to 71.4% for detecting Varroa destructor mites.

Voudiotis et al. [24] proposed a smart monitoring system for swarm detection. This system incorporates a new CNN engine designed to detect bees and notify apiarists in the event of swarming. The authors tested the performance and accuracy of several CNN models using both ARM- and GPU-based embedded systems. According to accuracy tests, the mAP metrics for the SSD MobileNetV1 and Faster R-CNN detectors were 42% and 70%, respectively.

Kongsilp et al. [25] developed a deep learning-based system that combines Mask R-CNN with a Kalman filter to track and segment individual honeybees in a beehive environment, achieving high performance at a frame rate of 10 fps. The system demonstrated a mAP of 0.85 for segmentation and 77% MOTA for tracking. It effectively handled dense and occluded scenarios, and its flexibility in segmentation and tracking shows promise for analyzing complex behaviors, such as honey bee dance patterns inside the hive.

### Monitoring by tracking bee flight paths

Nasir et al. [26] proposed a framework for recognizing invasive insects in unconfined flying conditions for smart beehives. The authors utilized a depth camera and a 3D coordinate system to present the acquired trajectories. They analyzed the trajectory lengths of individual insects and the time spent in front of the beehive. An artificial white background was employed to enhance the insect detection rate in the collected dataset. Multiple machine learning and deep learning models were used for classifying 3D trajectory and infrared data. This recognition framework demonstrated a classification accuracy of 97.1% for two types of hornets from the genus Vespa, as well as for honeybees.

Shimasaki et al. [27] investigated the activity of flying honeybees in a natural outdoor environment by analyzing the temporal frequency responses of brightness at the pixel level. The authors captured images with a resolution of 1024 × 1024 pixels using a 500 fps camera to obtain the wing-flapping frequencies of fast-flying honeybees. The results indicated that robust tracking can be achieved with activity sensing in the flapping frequency range of up to 250 Hz. Recently, the authors expanded their study by optimizing a bee detection model using the YOLOv8 architecture combined with an improved K-Nearest Neighbors algorithm, running at a resolution of 1920 × 1080 px and 1000 fps for real-time applications [28].

Williams et al. [29] developed optical and thermal camera systems to monitor beehive activity and track flight trajectories. The accuracy of bee tracking varied between 90% and 96%. To enhance the bee detection rate, additional boards were incorporated into the background of the camera’s field of view. Magnier et al. [30] investigated bee flight activity in front of the beehive using a white background and a camera positioned perpendicular to the landing board. They extracted bee flight paths by computing frame differences, utilizing edge detection in binary images, and applying an ellipse fitting algorithm for bee shape detection. Ratnayake et al. [31] developed an algorithm for individual honeybee monitoring and 2D track extraction among wildflower clusters. Their proposed background subtraction and segmentation algorithm yielded an 87% bee detection rate, compared to a detection rate of 61% achieved by a trained YOLOv2 model. Sun and Gaydecki [32] created a visual tracking system for reconstructing and analyzing the 3D flight trajectories of honeybees. The dataset was recorded using two calibrated cameras, resulting in an observable volume of over 200 m^3^. Flight track estimation relied on background subtraction, morphological transformation, bee contour detection, and Kalman filtering. Chiron et al. [33] employed stereo vision to detect and track honeybees at the entrance of the beehive, achieving a recovery rate of up to 80% for bee tracks in 3D space; however, accuracy decreased as the number of tracked targets increased. Rozenbaum et al. [34] developed a YOLOv5-based system to track honeybees in both 2D and 3D environments, operating at only 25 fps due to hardware limitations. Dropping every second frame allows for real-time processing but increases challenges in tracking the bees’ rapid movements across frames.

Two important conclusions can be drawn from recent efforts in vision-based monitoring systems for beehive entrances: first, CNN-based approaches are the most commonly used backbone detectors in most studies; second, there is a notable lack of analysis regarding bee behavior patterns at the hive entrance. Overall, recent studies have primarily focused on counting honeybees, analyzing traffic patterns, detecting pollen presence, and tracking bee posture. In this study, we propose algorithms to recognize bee behavior patterns on the landing board by analyzing spatial trajectories, speed of motion, and occurrence density maps of individual bees and groups.

## Materials and methods

### Overview

**Fig 1:**
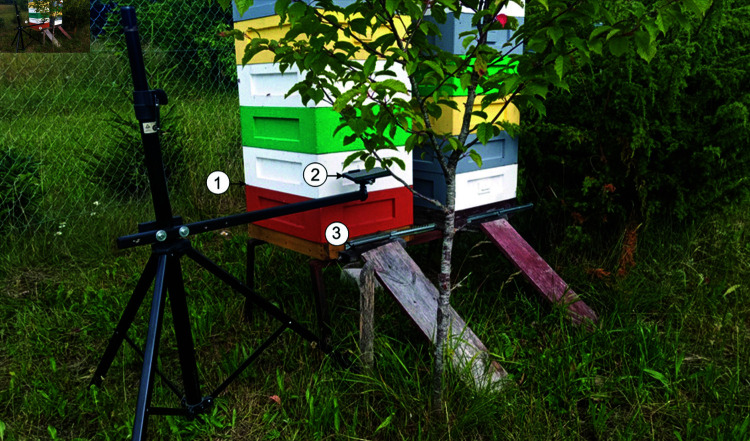
Data acquisition on local apiary. (1) Beehive, (2) portable camera for dataset collection at the hive entrance, (3) beehive landing board.

In this work, open-source tools were used to execute all stages of the bee behavior pattern recognition algorithm. To collect the bee image dataset, videos of hive landing boards were recorded at a local apiary in Vilnius district during June–July 2023 (Fig 1). The frames were captured from 30 cm above eight hive landing boards at 50 fps with a resolution of 1920 × 1080 pixels. The annotated dataset is publicly available [10]. Below is the list of software tools used in this work:

- *LabelImg* and *Labelme* tools were used to annotate bees for the detection and segmentation tasks, respectively.
- *Ultralytics* package was employed for training the YOLOv8 models for detection, and segmentation.
- *Supervision* package used to count and annotate bees within a specific zone.
- *BoT-SORT*, *ByteTrack*, *StrongSORT*, *DeepOC-SORT*, *OC-SORT* tracking algorithms applied to compare accuracy and speed in following individual bee movements.
- *TrackEval* framework used to evaluate multiple object tracking algorithms.

**Fig 2:**
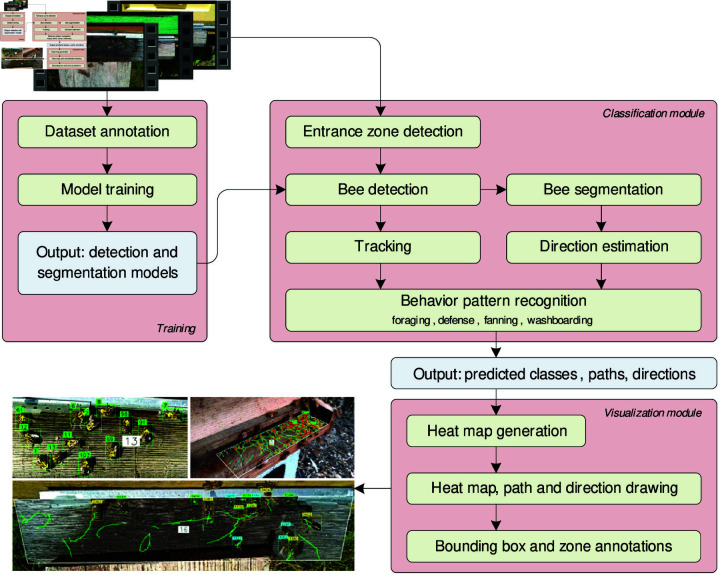
Conceptual algorithm of the bee behavior pattern recognition on the hive landing board.

To recognize and display the actions at the entrance ramp, behavior classification and visualization modules were developed. The classification module collects bee paths, estimates bee directions, recognizes behavior patterns, and outputs the predicted classes of bee behavior, paths, and directions for all detected bees in the zone (Fig 2). Custom code was created to capture the center points of detected bees and relate the coordinates of the bounding boxes to the frame indices for analyzing bee paths in 2D space. The object’s tracking function saves the center points of all detected objects, assigns IDs to the tracks, adds class information to each tracked object, saves the direction vector of each object, and associates all this information with the frame index for reconstruction during analysis. Associating data with the frame index provides easy access to any segment of the bee path. The bee paths and direction vectors are sent to the behavior recognition block, which detects defense, foraging, fanning, and washboard movements.

The optional visualization module was developed to generate occurrence density maps [35], draw bee paths and direction vectors, and merge all results into an output video file for visual presentation. The occurrence density map visualizes the concentration of bees on the landing board over a predefined period. Depending on the classified movement, individual bees or groups of bees (in the case of defense) are marked with colored bounding boxes. The paths, density maps, bee direction vectors, bounding box labels, a bee counter in the zone, and ramp borders can be added to the resulting frame as needed. The output frames can be live-previewed or stored as a sequence of images or as a video file.

### Detection

**Fig 3:**
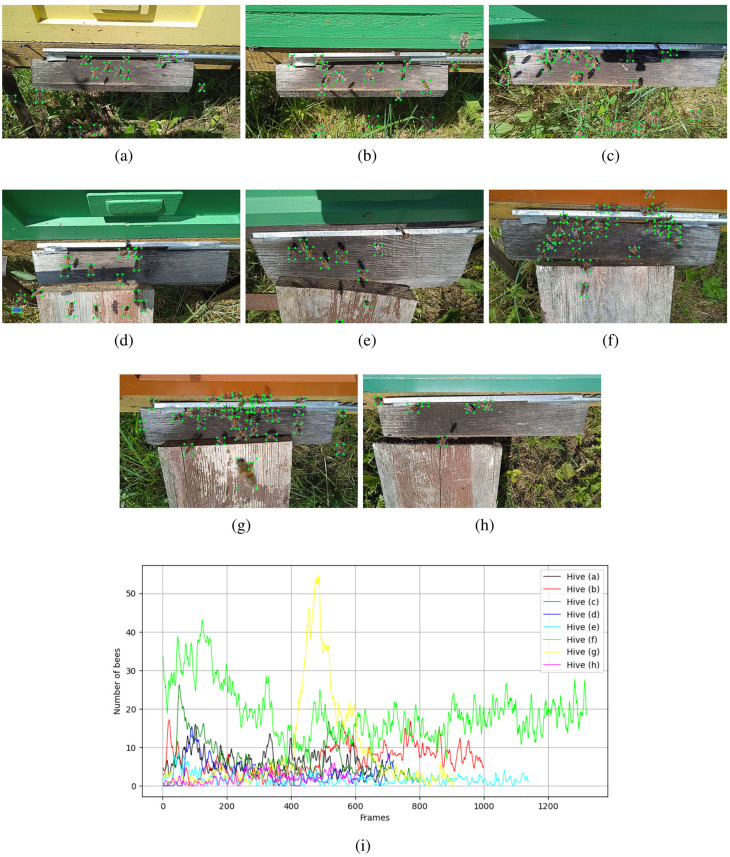
Images of 8 beehive entrances with annotated bees in the publicly provided bee detection dataset [10]. Images (a–h) represent different entrance ramps. Five hives (d–h) feature extended landing boards, used to facilitate bee landings. All the bees are assigned to one class, regardless of whether they are partially occluded, fully visible, blurred, or carrying pollen. Drones (g) are classified as bees. Number of annotated bees at the entrances of 8 beehives (i).

The bee detection dataset contains 7200 annotated frames. The annotations were saved in YOLO format. From the original recordings, frames were extracted at 0.2 second intervals and stored in JPEG format at a resolution of 1920 × 1080 px. The recordings were made on sunny and cloudy days from eight beehives. To speed up the labeling procedure, we manually annotated 5% of the images and trained the YOLOv8m model on this small dataset. The trained model was then applied to the full set of images to predict and save the bounding boxes for the bees. Subsequently, all labels were visually reviewed and corrected by the authors. During the creation of the dataset, all visible and partially occluded bees were labeled, regardless of their location in the images—whether on grass or above or below the entrance ramp (Fig 3). The graphs in Fig 3i show the number of annotated bees (mean over five frames) for each beehive. The peak in hive (g) (yellow curve) was caused by the beekeeper opening the hive’s top cover for inspection, resulting in more bees appearing on the entrance ramp.

For the current investigation, YOLOv8 models have been specifically trained for bee detection and function as single-class object detectors. If the CNN is trained for multi-class object detection, unwanted classes can be filtered out, retaining only those objects with confidence levels exceeding a user-defined threshold.

### Direction

**Fig 4:**
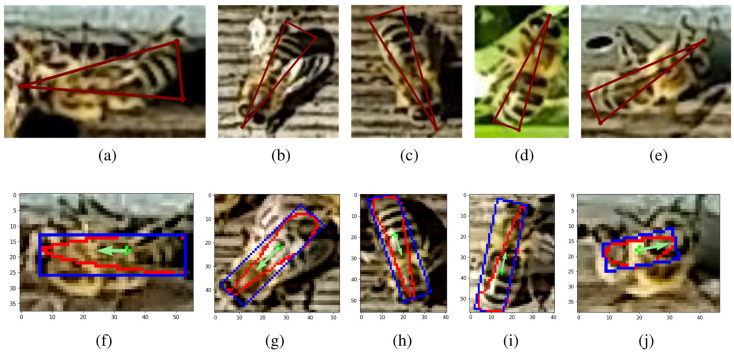
Triangle-annotated bees using the *Labelme* tool for direction estimation (a–e). Segmented bee contours (in red), minimum area rectangle (in blue), and bee direction vectors (in green) (f–j).

The segmentation model was trained on cropped images of bees to estimate their direction. It is used only when the user requires the computation of the bee’s direction; otherwise, segmentation is skipped to achieve a higher frame processing speed [36]. The segmentation dataset contains 1898 cropped images of bees. Each bee was annotated not with contours but with triangle-shaped markers, as shown in Fig 4a–4e. The most acute angle of the triangle indicates the direction of the bee.

**Fig 5:**
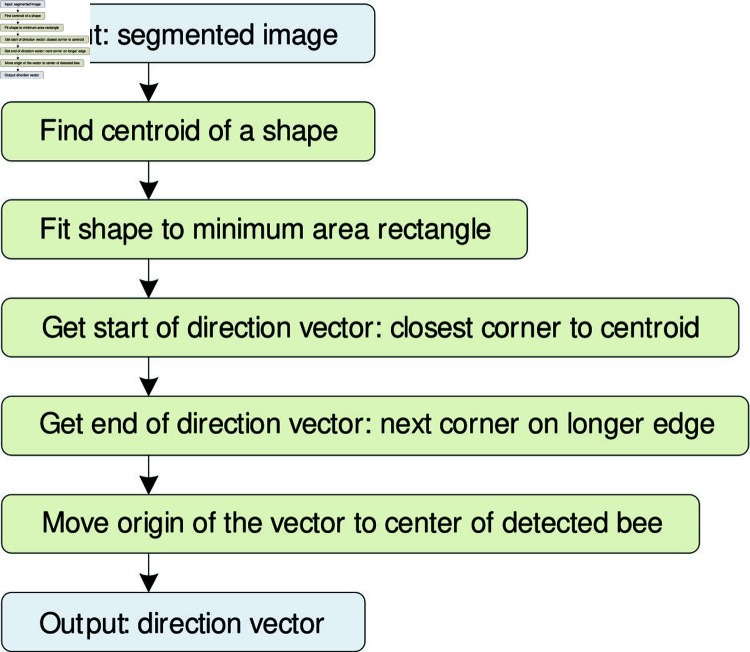
Conceptual algorithm of bee direction vector estimation based on segmentation.

The YOLOv8-seg model, after successful segmentation, produces a contour with a shape similar to the labeled triangle but with rounded corners (red contours in Fig 4f–4j). This contour is used as input for the conceptual algorithm that estimates the bee direction vector (Fig 5). The center of mass (centroid) of the red contour is computed, representing the arithmetic mean of all points in the shape. The bee contour (red) is then fitted to a minimal area rectangle (blue). We assume the bee direction vector is parallel to the longer edge of this rectangle. The closest corner to the center of mass is taken as the starting point of the direction vector, while the endpoint is the corner on the opposite edge of the rectangle. To visualize the direction of bees in the output file, the starting point of the direction vector is aligned with the center of mass, and the direction vector is inserted into the frame for each detected bee in the zone.

### Defense

Guard bees play a crucial role in defending the hive against intruders, such as robbing bees and wasps. Their presence and behavior are key indicators of the threat level to the colony. Increased defensive behavior can signal environmental stressors or threats that may necessitate beekeeper intervention [37]. The intensity and frequency of these defensive actions reflect the overall health of the colony. An aggressive response may suggest a stressed or weakened hive [8]. Therefore, analyzing behavior patterns over time is essential for identifying trends in defensive actions.

**Fig 6:**
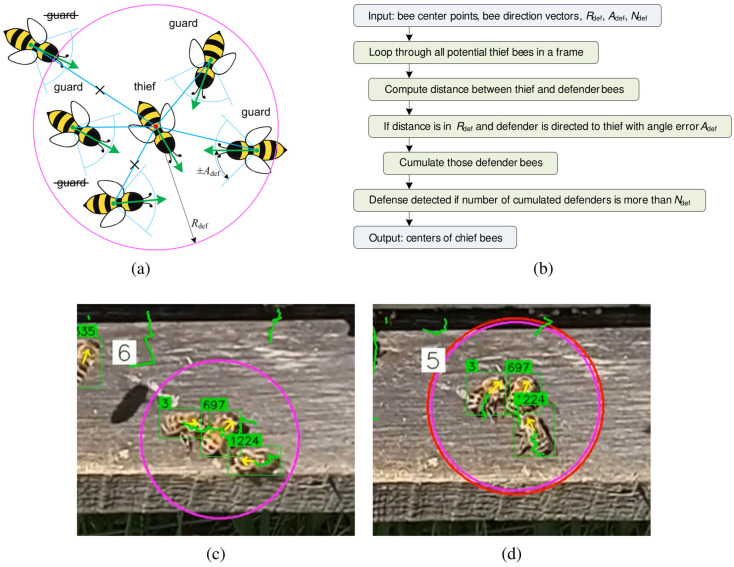
The principle of defense pattern detection (a). The conceptual algorithm for defense detection (b). The pink circle (c) marks the group of the thief and defenders after processing single frame with the defense detection algorithm. The red circle marks the defense pattern after post-processing the defense detections (d).

The proposed method for detecting defense patterns among guard bees is illustrated in Fig 6. During defense behavior, guard bees position themselves around an intruder (thief bee), facing it directly. The defense detection algorithm utilizes the center points and direction vectors of all detected bees within a monitored zone, along with three key parameters:

- $R_{\text{def}}$: Defense radius, defining how far defenders can be from the thief bee.
- $A_{\text{def}}$: Angle error, indicating the acceptable deviation between a defender’s direction and the direction to the thief bee.
- $N_{\text{def}}$: Minimum number of defenders required to confirm defense behavior.

The algorithm scans for potential thief bees in each frame, calculating distances to surrounding defender bees. Defense is recognized if a defender is within the radius $R_{\text{def}}$ and oriented towards the thief within the angle $A_{\text{def}}$. If the number of qualifying defenders exceeds $N_{\text{def}}$, defense behavior is confirmed, and the algorithm marks the defense area with a pink circle.

To address fluctuations in defense detection due to movement on the landing board, a post-processing algorithm tracks multiple bee groups involved in defense. This algorithm filters out short-term false positives and corrects false negatives using a moving average filter. Each tracked defense group is assigned an ID based on individual bee IDs over a specified time period ($T_{\text{def}}$). A stabilized red circle indicates a persistent defense pattern if it appears in at least 50% of the analyzed frames during this period.

**Fig 7:**
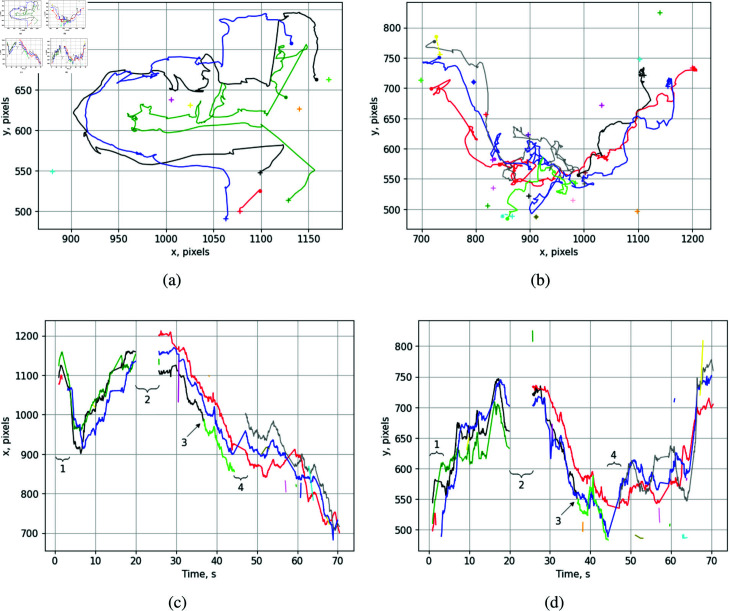
The representative 2D trajectories of honeybees while defending (a–b), the 2D distances in the x and y directions vs. time (c–d). The thief and two guard bees participated in the defense for the first 20 s, and for the next 45 s, during the period from 25 s to 70 s (c–d).

Fig 7a and 7b illustrate examples of honeybee trajectories during defense. The colored paths represent individual trajectories tracked with unique IDs, with starting points marked by "+" signs and endpoints indicated by dots. In these examples, two defender bees and one thief bee are consistently engaged in defense, resulting in prominent long trajectories. Additional bees present temporarily are initially assigned as defenders but are excluded from the defense pattern if they do not meet the specified angle and distance criteria.

Fig 7c and 7d illustrate the changes in the positions of the defense participants along the x and y axes over time. The first 20 seconds of these graphs correspond to the trajectories shown in Fig 7a, while the time span from 25 to 70 seconds relates to the paths depicted in Fig 7b. Four key incidents are marked during the tracking of the defense pattern. Mark 1 indicates that at the very beginning, the defense pattern was detected for one second. Then, a bee in the group temporarily disappeared for one second. Afterward, a new bee (the third bee) was assigned to the group with a new ID, causing a change in the path color from red to blue. Mark 2 shows where the defense pattern was completely lost for 5 seconds. However, from the 25th second onward, the same defense ID was reassigned to the pattern detected in the same location, as the center of the defense in the current frame was shifted by less than $R_{\text{def}}$. Mark 3 indicates a change in the ID of a bee, likely due to either a missed detection by YOLO or a loss of track by the tracking algorithm. Mark 4 refers to a lost defender for 3 seconds, which occurred due to overlap with another bee or a mismatch with the angle condition $A_{\text{def}}$. Subsequently, from 47 seconds to the end of the analyzed defense pattern, the third defender reappears and is shown with a gray path.

The presentation of defense patterns in the time domain demonstrates that the group of participants travels together on the landing board. Generally, the defense pattern lasts from several seconds to several minutes, depending on the number of participants and their defensive behavior. In most cases, fighting continues on the grass when all or part of the group falls off the landing board.

### Motion patterns

In this study, the zone of interest is restricted to the shape of the landing board; therefore, the investigation of motion patterns is conducted only within this entrance zone. Bee behavior patterns, such as foraging, fanning, or washboarding, can be detected by analyzing the speed of motion patterns of all bees tracked on the hive landing board.

#### Foraging.

Foraging behavior refers to the activities that bees engage in to locate, collect, and transport food resources, primarily nectar and pollen, back to the hive. Observing these behaviors can provide insight into the availability of local forage and the overall foraging efficiency of the colony [38]. Changes in foraging behavior can indicate shifts in environmental conditions, such as food shortages or variations in floral availability, which are critical to the sustainability of the hive [39].

**Fig 8:**
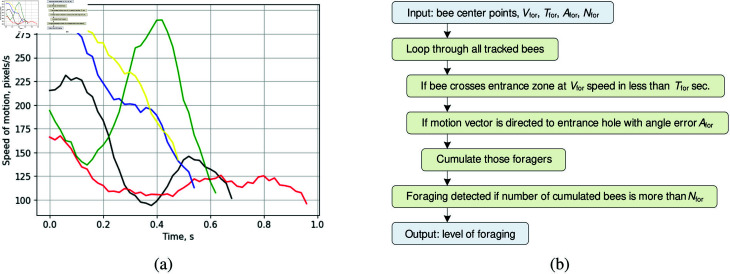
Speed of motion patterns on the hive landing board while foraging (a). Conceptual algorithm for foraging detection (b).

The speed of motion graphs for several foraging bees are presented in Fig 8a. Each colored curve corresponds to the motion speed of an individual bee over time. These patterns are captured on the landing board while monitoring the entrance zone, marked by the white polygon in Fig 11. Foraging behavior typically exhibits relatively high-speed and short-duration patterns. The speed of motion of the foraging bees varies in the range of 100 to 300 pixels/s, with appearances in the entrance zone lasting less than one second on average.

The proposed algorithm is designed to detect foraging bees arriving at the entrance of the beehive (Fig 8b). It utilizes the center points of all detected bees within a monitored zone and four key parameters:

- $V_{\text{for}}$: The minimum speed of motion for bees in the monitored zone.
- $T_{\text{for}}$: The time a bee can appear in the monitored zone on the landing board.
- $A_{\text{for}}$: The allowable angle error, defining how much the direction vector of a foraging bee can deviate from the direction toward the entrance hole.
- $N_{\text{for}}$: The minimum number of foragers required to confirm foraging activity.

The algorithm scans all tracked bees in each frame, checking their speed and time of appearance in the entrance zone. A bee is considered a forager if it crosses the entrance zone at a speed of at least $V_{\text{for}}$ within $T_{\text{for}}$ seconds, and if its motion vector is directed toward the entrance hole within the angle error $A_{\text{for}}$. Foraging behavior is confirmed when the number of detected foragers exceeds $N_{\text{for}}$. In this study, foraging activity is detected if at least one forager appears in the monitored zone.

#### Fanning.

Fanning is essential for cooling the hive, especially during hot weather. Monitoring this behavior can help assess whether bees effectively manage hive temperature and humidity, both of which are vital for brood development and overall colony health. Additionally, fanning plays a role in the distribution of pheromones, helping bees communicate about hive conditions and available resources [8].

**Fig 9:**
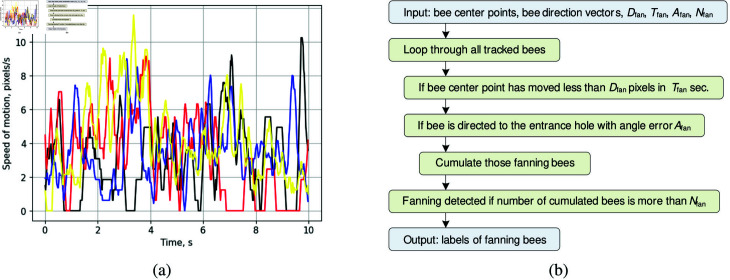
Speed of motion patterns on the hive landing board while fanning (a). Conceptual algorithm for fanning detection (b).

The proposed algorithm detects fanning behavior in bees, characterized by their stationary presence on the ramp for extended periods. The motion speed of fanning bees typically ranges from 0 to 10 pixels per second, resulting from slight fluctuations in the detected bounding boxes (Fig 9a). The fanning detection algorithm, illustrated in Fig 9b, processes the center points and direction vectors of all detected bees within a monitored zone, utilizing four key parameters:

- $D_{\text{fan}}$: The maximum distance a bee can travel during fanning.
- $T_{\text{fan}}$: The minimum time a bee must remain in the monitored zone to be considered part of the fanning pattern.
- $A_{\text{fan}}$: The allowable angle error, defining how much the direction vector of a fanning bee can deviate from the direction toward the hive entrance.
- *N*_fan_: The minimum number of fanning bees required to confirm the presence of fanning behavior.

The algorithm scans all tracked bees in the monitored zone. A bee is identified as part of the fanning pattern if it remains stationary for at least $T_{\text{fan}}$ seconds and is oriented toward the entrance hole within an angle error of $A_{\text{fan}}$. A bee is considered stationary if its center point travels less than $D_{\text{fan}}$ pixels during this time. Fanning behavior is confirmed when the number of detected fanning bees exceeds $N_{\text{fan}}$. The algorithm then labels these bees, updating their bounding boxes to cyan in the output frame (Fig 12e).

#### Washboarding.

While the exact purpose of washboarding is not fully understood, it is believed to be related to hive cleanliness and possibly the dislodging of pollen. This behavior facilitates traffic flow at the entrance of the hive and enhances surveillance for colony defense [40]. Observing washboarding can indicate the efforts of bees to maintain a healthy living environment.

**Fig 10:**
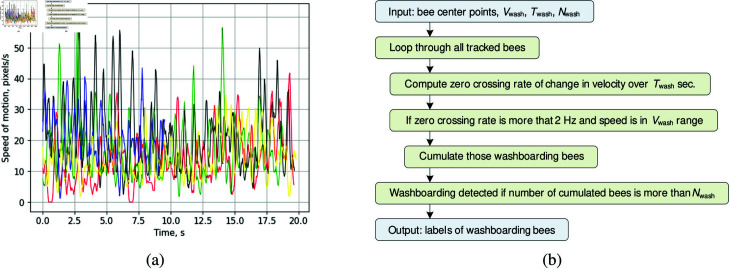
Speed of motion patterns on the hive landing board while washboarding (a). Conceptual algorithm for detecting washboard movement (b).

Washboarding behavior in bees is characterized by a periodic forward-backward movement as they scrape the wooden surface of the landing board after foraging [41]. This behavior exhibits noticeable periodicity in speed patterns (Fig 10a), with motion speeds ranging from 0 to 60 pixels per second. The developed washboard movement detector (Fig 10b) tracks the periodicity and amplitude of bee speed. It processes the center points of all detected bees in the monitored zone, utilizing three key parameters:

- $V_{\text{wash}}$: The range of average speeds used for detecting washboarding patterns.
- $T_{\text{wash}}$: The duration required for the washboarding movement to be continuously repeated.
- $N_{\text{wash}}$: The minimum number of washboarding bees needed to confirm the behavior.

The algorithm scans all tracked bees in the monitored zone and calculates the zero-crossing rate of change in bee velocity (acceleration). Washboarding is identified if the zero-crossing rate exceeds 2 Hz, is continuously repeated for at least $T_{\text{wash}}$ seconds, and if the average speed falls within the range defined by $V_{\text{wash}}$. The pattern is confirmed when the number of detected washboarding bees exceeds $N_{\text{wash}}$. The algorithm then labels these bees, updating their bounding boxes to orange in the output frame (Fig 12f).

### Summary

The activity recognition method relies on several parameters that are outlined in Table 1. The rationale behind these parameters is rooted in the empirical analysis of bee behavior, as derived from annotated datasets and behavioral observations. Each parameter has been chosen based on its relevance to the specific behavioral patterns being identified.

**Table 1:** Parameters used in the activity recognition algorithms.

Foraging	Fanning	Washboarding	Defense
$V_{\text{for}}>100$ pixel/s	$D_{\text{fan}}<10$ pixel	$V_{\text{wash}}<60$ pixel/s	$R_{\text{def}}\leq 2$ bee lengths
$T_{\text{for}}>0.1$ s	$T_{\text{fan}}>1$ s	$T_{\text{wash}}>2$ s	$T_{\text{def}}>1$ s
$A_{\text{for}}=\pm 60^{\circ }$	$A_{\text{fan}}=\pm 90^{\circ }$	zero-cross rate > 2 Hz	$A_{\text{def}}=\pm 45^{\circ }$
$N_{\text{for}}\geq 1$	$N_{\text{fan}}\geq 1$	$N_{\text{wash}}\geq 1$	$N_{\text{def}}\geq 2$

Parameters such as speed thresholds (e.g., $V_{\text{for}}>100$ pixel/s for foraging) and time duration thresholds (e.g., $T_{\text{for}}>0.1$ s for foraging) are derived from observed bee behavior patterns. These thresholds ensure that behaviors like foraging (quick movements toward the entrance) are accurately distinguished from other behaviors. Similarly, fanning is characterized by minimal movement ($D_{\text{fan}}<10$ pixel over $T_{\text{fan}}>1$ s), while washboarding is detected through periodic movement patterns within speed and frequency ranges. Parameters like $A_{\text{for}}=\pm 60^{\circ }$ and $A_{\text{fan}}=\pm 90^{\circ }$ provide angle error margins that account for variations in bee orientation relative to the hive entrance. These values were likely chosen based on empirical observations of natural variations in bee movements during specific activities. Defense detection incorporates parameters such as the defense radius ($R_{\text{def}}\leq 2$ bee lengths), angle error ($A_{\text{def}}=\pm 45^{\circ }$), and the number of defenders ($N_{\text{def}}\geq 2$) required for confirmation. These parameters align with the spatial clustering behavior observed in defensive bees surrounding a threat. Parameters such as $N_{\text{for}}\geq 1$, $N_{\text{fan}}\geq 1$, $N_{\text{wash}}\geq 1$, and $N_{\text{def}}\geq 2$ ensure that behaviors are detected only when a sufficient number of bees exhibit the characteristic patterns.

## Results and discussion

This section addresses the following topics in succession: dataset preparation and training; performance comparison with state-of-the-art approaches; performance comparison across different detection models and tracking algorithms; evaluation of average time per algorithm stage; analysis and visual presentation of occurrence density maps, tracks, and detections; and classification of behavior patterns.

### Bee detection

Typically, apiarists use very similar ramps in all hives within an apiary. However, the color, shape, and size of the ramps, entrance holes, and hives can vary significantly between different apiaries. For this study, we used a dataset recorded from several beehives within a single apiary to capture various patterns. This approach was chosen because some behaviors may be observable at one entrance but not at another, depending on the strength of the colony [8]. The recognition of bee behavior in this study is based on the track analysis of individual bees at the entrance to the beehive. At this stage of monitoring system development, the goal is to train the model for accurate bee detection, as this significantly influences the precision of the tracking algorithm. The YOLOv8 detection models were trained on 6000 frames and tested on 1200 frames. The data split was performed randomly, with 5/6 of the frames assigned to the training set and 1/6 to the test set. Thus, during both training and testing, the model encountered images from the same hive but with different placements of bees at the entrance.

The input image size for the trained model was set to 640 × 384 pixels. YOLOv8 models were trained using an RTX 2060 GPU, requiring fewer than 100 epochs to achieve minimal training loss, with a batch size of 8 for each detection model. During training, optional augmentation parameters were applied to enhance the model’s robustness and improve generalization to unseen data. Specifically, the scale gain was set to 0.5 to simulate bees at varying distances from the camera, and the probability of flipping images left-to-right was set to 0.5 to increase dataset diversity and help the model learn symmetrical objects.

**Table 2:** Comparative evaluation of the proposed implementation to state-of-the-art methods for bee detection in images.

Authors	Implementation	Dataset	Resolution	FPS	Accuracy
Tu et al. [18]	BS and statistical analysis	–	1920 × 1080	0.26	98
	on Raspberry				
Chiron et al. [33]	Stereovision on CPU	- ∕ 500	752 × 480	10	80
Voudiotis et al. [24]	SSD + MobileNetV1 on CPU	6527 ∕ 100	300 × 300	11	42
	Faster R-CNN on CPU		600 × 600	4	70
Ngo et al. [12]	YOLOv3-tiny on GPU	3000 ∕ 500	640 × 480	25	94
Bilik et al. [23]	YOLOv5 on GPU	561 ∕ 115	640 × 640	30	95
	SSD+VGG16 on GPU			1	92
	SSD+MobileNetV2 on GPU			9	51
Majewski et al. [2]	Mask R-CNN + ResNet50	143 ∕ 37	1920 × 1080	–	95
Kulyukin and Mukherjee [16]	Custom ConvNet on GPU	167261	32 × 32	–	94
This study	YOLOv8n on GPU			57	92
	YOLOv8s on GPU			49	96
	YOLOv8m on GPU	6000 ∕ 1200	1920 × 1080	36	97
	YOLOv8l on GPU			20	98
	YOLOv8x on GPU			15	98

YOLOv8 offers five pre-trained models for object detection tasks, each retrained on our dataset to evaluate how model speed and accuracy vary with complexity. Table 2 presents the mean accuracy and speed of these five YOLOv8 models, listed in ascending order of complexity, specifically for the bee detection task. The "Dataset" column indicates the number of images used for training and testing the detector. As the complexity of the network increases, precision improves, rising from 92% for the YOLOv8 nano model to 98% for the extra-large model. However, this increase in accuracy comes at the cost of processing speed, which decreases from 57 fps to 15 fps as the number of parameters increases.

All the state-of-the-art methods listed in Table 2 use their own datasets, which range from a few hundred to several thousand images. Ngo et al. [12] use a monotone black background, while Tu et al. [18] use a monotone white background to improve contrast and enhance the detectability of bees at the hive entrance. Other studies utilize native entrance ramps [2,16,33] or native honeycombs [23,24] as backgrounds when tracking bees inside the hive. Most of the state-of-the-art methods for bee detection in images, as cited in Table 2, are based on CNN implementations. For instance, Chiron et al. [33] detect bees in 3D space using stereovision and then reconstruct bee tracks. Tu et al. [18] use background subtraction (BS) and statistical analysis to count bees in the frame, while Kulyukin and Mukherjee [16] detect motion in 1920 × 1080 px frames and then apply a custom configuration of ConvNet to 32 × 32 px cropped images. According to the latest approaches, bee detection accuracy in images varies up to 98%.

### Direction estimation

The extensive results of training different YOLOv8 models are presented in Table 3. All initial models were trained in fewer than 100 epochs. Two approaches were tested to estimate the direction of the bees. In the first approach, nano-, small-, and medium-sized detection models were used to locate bees in the frame. Then, for each detected bee, the smallest YOLOv8n-seg segmentation model was applied to estimate the direction of the bee in the cropped image. In the second approach, the three largest segmentation models were used to detect and estimate directions simultaneously for all bees in the frame. These models provide box and mask precision metrics to evaluate detection and segmentation outputs. The best box precision, 98%, is achieved with the YOLOv8m model, which will be used in the bee behavior pattern detection algorithm. Meanwhile, the smallest YOLOv8n-seg segmentation model is used for estimating bee directions, achieving 98% mask precision in the segmentation of triangle-shaped bees. The YOLOv8n-seg segmentation model works in conjunction with the YOLOv8m bee detection model. YOLOv8n-seg receives detections (cropped images of bees) from the YOLOv8m model, resizes them to 64 × 64 pixels, and estimates the direction of the bee more precisely than using a single YOLOv8m-seg model for both bee detection and segmentation in the full frame. The YOLOv8m-seg yields 97% box precision and only 83% mask precision, which is 1% worse for detection and 15% worse for segmentation compared to the YOLOv8m and YOLOv8n-seg models, respectively. From a precision perspective, it is preferable to use separate models: YOLOv8m for bee detection and YOLOv8n-seg for direction estimation. However, using two models takes more time than using a single model. For comparison, even more complex models, such as the large YOLOv8l-seg and extra-large YOLOv8x-seg, do not show reliable mask precision for bee segmentation, achieving only 84% and 86%, respectively. Bozek et al. [42] achieved a comparable precision of 82% in segmentation for bee orientation estimation using a U-Net model on an original dataset labeled by the authors.

**Table 3:** Training results of the YOLOv8 models used in bee detection and direction estimation.

Approach	Initial model	Precision	Recall	mAP50	mAP50-95	Parameters	ms/ inference	Input size
1	YOLOv8n	Box: 0.93	0.88	0.92	0.59	3.2M	9.4	
	YOLOv8s	Box: 0.96	0.94	0.96	0.63	11.2M	11.3	640 × 384
	YOLOv8m	Box: 0.98	0.97	0.97	0.65	25.9M	16.4	
	YOLOv8n-seg	Mask: 0.98	0.98	0.97	0.44	3.4M	9.0	64 × 64
2	YOLOv8m-seg	Box: 0.97	0.92	0.97	0.59	27.3M	20.9	
		Mask: 0.83	0.76	0.81	0.29			
	YOLOv8l-seg	Box: 0.96	0.91	0.97	0.58	46.0M	37.4	640 × 384
		Mask: 0.84	0.78	0.81	0.30			
	YOLOv8x-seg	Box: 0.97	0.93	0.97	0.58	71.8M	56.3	
		Mask: 0.86	0.78	0.83	0.32			

### Tracking

The tracking dataset includes annotated tracks of bees during foraging, defense, fanning, and washboarding. It contains 17162 tracks visible in 21946 frames, which corresponds to 438 seconds of footage. The tracks are annotated only for the bees on the landing board. Each tracked bee is annotated with the following information: [*frame-id*, *track-id*, *bb-left*, *bb-top*, *bb-width*, *bb-height*]. The coordinates of the bounding box are normalized to the width and height of the frame. The MP4 recordings, annotations, and entrance zone coordinates are publicly available [10].

Here, general metrics (MOTA, HOTA, and IDF1) [43] were used to evaluate multiple object tracking algorithms (Table 4) employing the *TrackEval* framework. According to MOTA, the best accuracy was achieved with BoT-SORT and ByteTrack. However, the HOTA metric favors BoT-SORT, StrongSORT, and Deep OC-SORT. Deep OC-SORT outperforms the other tracking algorithms in terms of the IDF1 score. ByteTrack and OC-SORT provide the shortest update times for the tracker, as these tracking algorithms do not use appearance modeling [44]. Therefore, ByteTrack or OC-SORT should be considered for real-time applications, as they introduce 10 to 20 times less latency in tracking bees compared to BoT-SORT, StrongSORT, or Deep OC-SORT.

**Table 4:** Comparison of tracker accuracy and update time across different tracking algorithms applied to 1920 × 1080 video with a detector confidence threshold of 0.3. Tracker update time is measured in milliseconds per frame.

Metric	BoT-SORT	ByteTrack	StrongSORT	Deep OC-SORT	OC-SORT
MOTA	0.79	0.79	0.77	0.78	0.77
HOTA	0.64	0.62	0.64	0.64	0.63
IDF1	0.79	0.76	0.78	0.80	0.77
Tracker update time, ms	21.0	1.7	27.3	34.3	2.1

Table 5 presents the average time required to process the most time-intensive stages of the algorithm (Fig 2). Loading an image into the YOLOv8m model and applying zone and unwanted class filtering takes an average of 27 ms per frame. Running the YOLOv8n-seg model on cropped bee images for direction estimation requires 9 ms per bee. Alternatively, using the YOLOv8m-seg model as a joint approach for detection and segmentation takes 41 ms per frame, eliminating the need to run an additional model on cropped images for direction estimation. However, this approach results in a 15% reduction in mask segmentation precision compared to the YOLOv8n-seg model. Additionally, extracting the bee’s direction from the segmented contour requires an extra 64 μs per bee. Bee tracking with ByteTrack requires 1.7 ms per frame, path drawing takes 1.8 ms per frame, and computing the occurrence density map requires 7.3 ms per frame. The drawing stage, which takes 460 ms per frame, is independent of the selected detection model, as it operates with the center points of the detected bees.

**Table 5:** Average time per algorithm stage. In the first line, YOLOv8m is used for bee detection in 1920 × 1080 px frames, and YOLOv8n-seg is applied for bee segmentation in 64 × 64 px cropped images. In the second line, YOLOv8m-seg is used for both bee detection and segmentation.

CNN model	Detection	Tracking	Paths	Direction	Density map	Map
			**drawing**	**estimation**	**computation**	**drawing**
YOLOv8m & YOLOv8n-seg	27 ms/frame	1.7 ms/frame	1.8 ms/frame	9 ms/bee	7.3 ms/frame	460 ms/frame
YOLOv8m-seg	41 ms/frame			64 *μ*s/bee		

### Visualization

**Fig 11:**
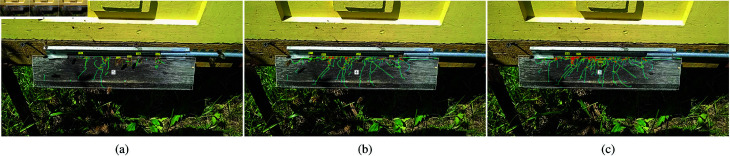
Occurrence density maps of the bee tracks on the landing board after 5 s (a), 10 s (b), and 20 s (c) of monitoring the entrance zone.

**Fig 12:**
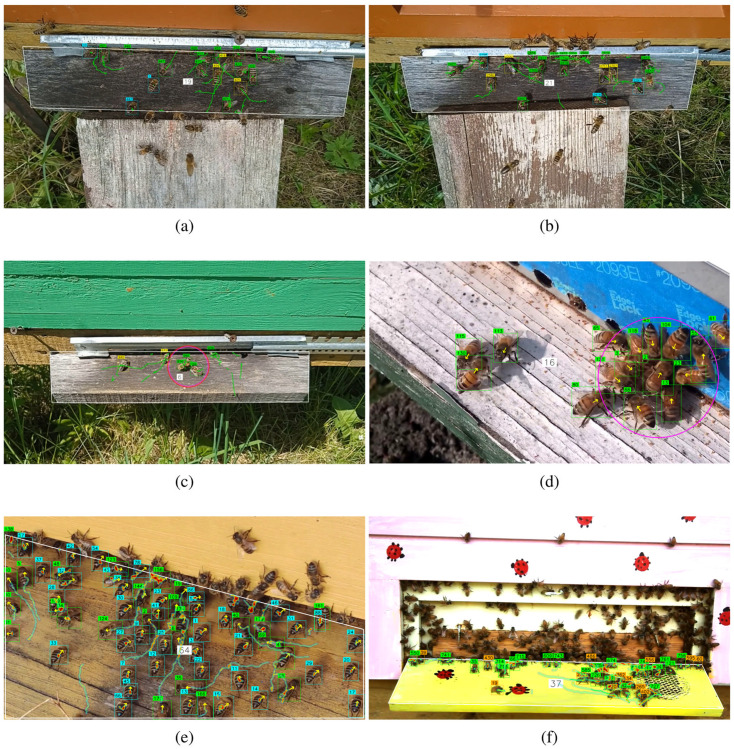
Detected bees on the landing boards. Foraging bees are annotated in yellow (a–c), with green tracks showing the paths of the bees during the last second. The pink circle marks the detected defense event (c, d). Fanning bees are marked with cyan bounding boxes (a, b, e). Bees exhibiting washboard movement are annotated in orange (f), and occurrence density maps of the last two seconds are captured within white polygon-constrained zones (e, f). Optional direction vectors are marked in yellow (d, e).

The proposed algorithm for recognizing bee behavior patterns was tested on videos collected from hive entrances, as well as on recordings shared online by other beekeepers. The analysis of bee movements relies heavily on the accuracy of the bee tracks, which are highly sensitive to camera motion. Therefore, all selected videos were captured using stationary cameras mounted above the hive landing board. The analyzed recordings include various bee behavior patterns, such as foraging, defense, fanning, and washboarding. Since landing boards can vary between different beehives, the bee detection zone was manually adjusted before processing each video file. Figs 11 and 12 display frames with tracked bees in designated on-demand detection zones.

The presence of bees on the landing board of the hive can be represented as a density map of accumulated bee paths over a specified period. The detected bees, along with their traveled paths and the density map, are illustrated in Fig 11. The monitored entrance zone is defined by a configurable polygon that covers the landing board. A bee counter is displayed at the center of this polygon and can optionally be included in the resulting frame. Graphical elements such as bee indices, markers, trajectory paths, direction vectors, and occurrence density maps can be added to the frame as needed. Users can also adjust the amount of historical data displayed in the trajectories and density maps, allowing for a backward view from the current frame. Fig 11 depicts the density maps after 5 seconds, 10 seconds, and 20 seconds of monitoring the entrance zone during foraging. These maps illustrate traffic intensity in areas where bees predominantly cross the landing board. Separate density maps can be generated for incoming or outgoing traffic, or even for bees exhibiting specific behavior patterns. During foraging, bees quickly traverse the entrance zone, resulting in a relatively higher number of straight paths. Over time, these paths overlap to create a saturated map during longer analysis periods.

Fig 12 presents frames showing bees detected under different behavior patterns on the landing board of the hive. In Fig 12d, bees are tracked throughout the entire area visible to the camera, while in the other figures, detections are limited to polygon-constrained zones. In this study, defense behavior is identified by recognizing specific constellations of bees. The defense pattern can also be classified as an additional object class by training a CNN to detect the region where a group of defenders surrounds a thief bee. In future work, we will explore a context understanding approach [45], which involves detecting groups of bees as an additional class rather than identifying individual bees. This method will allow us to pass coordinates along with direction vectors through the proposed defense detection algorithm.

The hot spots on the occurrence density map (Fig 12e) from the last two seconds of monitoring indicate that the bees are stationary for fanning. These bees are marked with cyan boxes, and arrows indicate that they are facing the entrance hole. The fanning pattern can sometimes be misclassified as bees resting on the landing board. Typically, bees fan in groups with a distinctive pose, facing downward toward the entrance with their tails raised. It may be beneficial to annotate groups of fanning bees within a common bounding box or to detect their distinctive pose when viewed from above. Fanning can also be identified by detecting flapping wings on sunny days. Bees exhibiting washboarding movement are labeled with orange boxes (Fig 12f). The occurrence density map shows that washboarding bees are characterized by slight local movements.

### Behavior recognition

**Fig 13:**
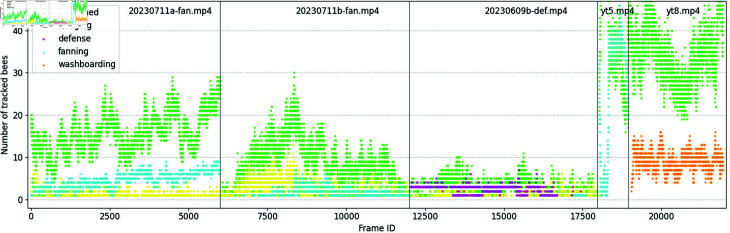
The distribution of the activities in the behavior classification dataset. Horizontal axis presents frame index, vertical axis shows number of tracked bees assigned to one of the four behavior classes. Vertical lines separate five different records. The corresponding annotated frames with detected activities in these records are presented in Fig 12a, 12b, 12c, 12e, 12f, respectively.

The proposed behavior recognition algorithms are based on the analysis of the orientation, path, and speed of tracked bees. Consequently, the behavior classification dataset includes annotated tracks of bees across four classes: foraging, defense, fanning, and washboarding. Bees are annotated in the same format as in the tracking dataset, with each object instance represented on a single line. This line includes four flags for behavior classes at the end: [*frame-id*, *track-id*, *bb-left*, *bb-top*, *bb-width*, *bb-height*, *class-for*, *class-def *, *class-fan*, *class-wash*]. For example, in the case of foraging, the class vector is annotated as [1, 0, 0, 0], indicating that the tracked bee in the current frame is classified as forager. To accelerate the labeling process, the classes of bee tracks were predicted using the developed behavior detection algorithms and subsequently reviewed by the authors. The dataset is publicly available and contains 17162 tracks visible across 21946 frames [10]. Fig 13 illustrates the class distribution across five annotated records. The names of these records correspond to the files provided in the dataset repository. The distribution of bee behaviors varies across the five video records, with foraging and fanning most prominent in the first (20230711a-fan.mp4) and second videos (20230711b-fan.mp4), while defense is concentrated in 20230609b-def.mp4 and washboarding is primarily observed in yt8.mp4. Undefined activities are consistently significant across all videos. This variability reflects the situational nature of the behaviors, with each video highlighting specific dominant activities.

**Fig 14:**
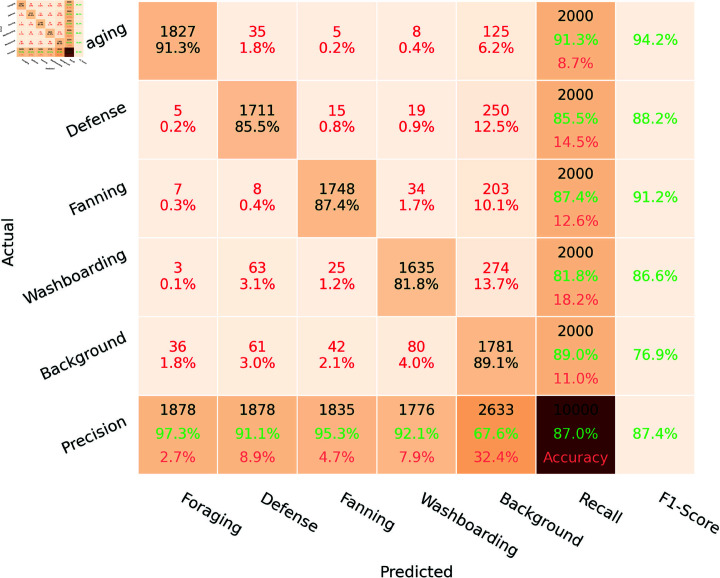
Confusion matrix for bee behavior pattern recognition.

The performance of the algorithms developed for behavior pattern recognition is summarized in the confusion matrix (Fig 14). This matrix illustrates the interrelationships among the four behavior patterns. Recognition is performed at the frame level, utilizing 2000 frames for each behavior from the dataset. When two behaviors occur simultaneously, both behavior classes are marked as true positives. For example, if one bee is fanning while another is foraging, that frame will be assigned to both classes. An additional background class is included in the matrix. Frames without bees, frames where bees are resting on the landing board, or frames where bees are moving randomly are assigned to the background class. The sixth row and sixth column of the matrix represent precision and recall metrics, respectively. The intersection of these precision and recall series indicates the overall accuracy of the proposed algorithm. The upper value in each cell represents the number of frames assigned to a specific class. The results indicate that foraging patterns are more robust compared to the other behaviors. The high precision of foraging detection reflects a low rate of false positives in the first column because the foraging pattern is associated with high-speed motion of bees (above 100 pixels/s), while other pattern detectors operate below this speed threshold. Recall indicates that the actual foraging pattern is correctly predicted in 91% of frames. The 9% recall error is attributed to cases where bees, after landing on the board, do not immediately move toward the entrance hole; instead, they may linger at the entrance, cross the board at lower speeds along nonlinear paths, or land on the front wall of the hive above the entrance—outside the monitored zone—before traveling to the entrance hole.

In general, all tested patterns are more frequently confused with the background pattern than with each other. During pattern classification, if foraging, fanning, washboarding, or defense patterns are not recognized as true positives in the processed frame—due to inaccuracies in the pattern recognition algorithms, lost trackers, or missed detections—that frame is predicted as background. Consequently, the precision for the background is 67%, which is relatively lower compared to the other patterns. The recall indicates that the actual background video is predicted as the true class with an 89% probability. The actual background pattern resembles defense, fanning, and washboarding more closely than it does foraging. Fanning is often misidentified as the background pattern because some bees can rest on the landing board without moving and may be oriented toward the entrance hole. The defense pattern can also be misclassified as background because small groups of bees sometimes appear on the landing board, such as during pheromone dispersal at the hive entrance to guide young bees back to the hive or when guard bees patrol the landing board to inspect incoming bees. Additionally, small groups of bees may interact with each other, groom one another, or communicate through various behaviors.

The robustness of the proposed framework is inherently influenced by the sequential nature of its processing steps, where the quality of one stage can significantly impact subsequent stages. For instance, tracking algorithms are crucial for maintaining continuity in identifying individual bees but may encounter challenges due to fast movements and similar appearances among bees, resulting in frequent ID switches. These ID switches disrupt the continuity of bee trajectories, leading to inaccurate estimations of movement speed and direction—both vital for behavior classification. Furthermore, tracking accuracy is directly influenced by the precision of the bee detection model; any missed or false detections can result in breaks in tracking or incorrect trajectory assignments. Similarly, the orientation estimation step heavily depends on the precision of the segmentation model; inaccuracies in segmentation can lead to errors in direction estimation. Lastly, the precision of activity recognition relies on the parameter settings of the algorithms used to identify the four behavior classes. For example, thresholds for speed, angle, and duration directly impact the classification of foraging, fanning, washboarding, and defense behaviors. Future work should investigate ways to optimize these interconnected steps by improving detection and segmentation accuracy, enhancing tracking algorithms to reduce ID switches, and refining parameter tuning to increase the overall robustness of the framework.

### Future work

To the best of our knowledge, there is currently no open-source annotated dataset available that comprehensively covers all the bee behavior patterns investigated in this work. Our next step will be to extend the current dataset to include various abnormal behaviors, focusing on swarm preparation, defense, and robbing. These behaviors are of particular interest to beekeepers; however, they are relatively rare, necessitating longer video recordings to capture such instances. Early detection of robbing can prevent nectar loss, while predicting swarming times allows for effective colony management before the old queen leaves to find a new nesting location outside the apiary.

To achieve this, we plan to utilize a camera mounted above the bee landing board, directed toward the hive entrance. In our current study, prediction was performed on a single class of objects, achieving 98% detection and segmentation performance. This relatively high precision is attributed to the robust patterns exhibited by the bees themselves. Furthermore, it would be beneficial to include additional classes of insects, such as drones, wasps and hornets, to detect them at the hive entrance. Incorporating these insects into the dataset would enhance the accuracy of classifying different insect behaviors at the hive entrance. In general, the proposed algorithm can be adapted to track individual insects and investigate behavior patterns of any insect species in the wild by analyzing their tracks and specific constellations.

Future research will focus on collecting datasets that expand bee behavior classes, particularly preparation for swarming and robbing—behaviors that require beekeeper intervention. Additionally, future work should include an analysis of bee behavior patterns at the hive entrance, along with occurrence density maps and bee tracking in multiple independent zones above and below the landing board.

## Conclusion

In our study, we proposed a conceptual algorithm to recognize the behavior patterns of bees at the entrance to the beehive. This algorithm involves bee counting, tracking, direction estimation, pattern detection, and the generation of an occurrence density map within a user-defined zone of interest. The YOLOv8 models provide a reliable solution for detecting and tracking individual bees engaged in activities such as foraging, fanning, washboarding, and defending. Speed-of-motion graphs can be utilized to identify these behavior patterns. However, during intensive foraging, swarming, or defense activities, bees tend to mix rapidly, leading to partial occlusion. As a result, some detections may be lost or mistakenly assigned to the nearest bee. Differentiating between behaviors such as intensive foraging, swarming, and guarding presents a significant challenge that requires a more extensive dataset and in-depth investigation.

The trained YOLOv8m model achieves 98% mean precision in bee detection and can process up to 36 fps, making it suitable for real-time applications. This detection speed outperforms state-of-the-art approaches, while the accuracy is comparable and largely depends on the selected dataset used for investigation. ByteTrack provides the fastest tracker update rate at 1.7 ms per frame, meeting the demands of real-time scenarios. The 87% accuracy in recognizing foraging, fanning, washboarding, and defense patterns makes the proposed system suitable for monitoring hive conditions.
